# Computed Tomography Analysis of the Feline Infraorbital Foramen and Canal

**DOI:** 10.3389/fvets.2020.619248

**Published:** 2021-01-27

**Authors:** Lily V. Davis, Naomi K. Hoyer, Pedro Boscan, Sangeeta Rao, Jennifer E. Rawlinson

**Affiliations:** ^1^Colorado State University, Fort Collins, CO, United States; ^2^College of Veterinary Medicine and Biomedical Sciences, Colorado State University, Fort Collins, CO, United States; ^3^Epidemiology and Biostatistics, Department of Clinical Sciences, CVMBS, Colorado State University, Fort Collins, CO, United States

**Keywords:** computed tomography, feline, infraorbital canal, maxillary nerve block, regional anesthesia

## Abstract

Feline skull anatomic variation is plain to see with casual observation. Obtaining an in-depth understanding of this anatomic variability is critical to performing safe and effective regional anesthesia for dental procedures and maxillofacial surgeries. Maxillofacial anatomic variability is proven to impact the placement and efficacy of nerve blocks in dogs and horses, but similar studies have not been performed in cats. This study's main objective was to evaluate the anatomy of the infraorbital foramen and canal in relation to regional anatomic landmarks in brachycephalic and mesaticephalic cats. Significant anatomic variability was identified, particularly among cats with brachycephalic skulls.

## Introduction

Regional nerve blocks are a critical component of analgesia and anesthesia for feline patients undergoing dental extraction and oral surgery. Regional nerve blocks have been shown to reduce the minimum alveolar concentration (MAC) of gas anesthetics, improve anesthesia management and decrease pain (1–8). Accurate placement of local anesthetics for regional anesthesia is necessary to obtain effective analgesia and minimize the occurrence of complications, which include hematoma formation and trauma to nearby structures, particularly the eye (9–12). Trigeminal nerves provide sensory innervation to the tissues of the oral cavity and face. The maxillary nerve is a branch of the sensory root of the trigeminal nerve. It emerges from the rostral alar foramen into the pterygopalatine fossa and runs parallel with the maxillary artery on the surface of the medial pterygoid muscle. As it enters the maxillary foramen (the caudal border of the infraorbital canal), it becomes the infraorbital nerve continuing rostrally through the infraorbital canal and exiting through the infraorbital foramen (dorsal to the maxillary fourth premolar tooth). The caudal and middle superior alveolar branches exit the maxillary nerve in the pterygopalatine fossa in cats (13–15). In order to administer the most effective maxillary regional anesthesia for dental procedures, these branches must be anesthetized by the block.

Anatomic variability has been established in a variety of species, including humans (16). In dogs, there are a number of publications examining anatomical variation, placement and effectiveness of regional blocks and nerves in the maxillofacial region (17–19). Evaluations of canine and equine maxillary nerves and infraorbital canals have identified anatomic variability that may affect regional anesthetic placement (20, 21). This is a retrospective descriptive study to critically evaluate the anatomic location, size, and shape of the infraorbital foramen and canal in cat skulls using computed tomography (CT). Beyond the main objective of the study, additional aims included describing the location of these bony structures in relation to vital adjacent anatomy and comparing mesaticephalic and brachycephalic skull types. It was hypothesized that there would be individual variability in the anatomy of the infraorbital canal and foramen, significantly so in brachycephalic cats when compared to mesaticephalic cats.

## Materials and Methods

The study design was reviewed by the Colorado State University Clinical Trials Review Board and because the study used retrospective computed tomographic imaging no approval was required by the Institutional Animal Care and Use Committee. All clients signed a general research release of clinical data upon admission of their animals to the hospital. Forty-two CT scans of feline heads were evaluated. The images were obtained from Colorado State University's James L. Voss Veterinary Teaching Hospital picture archive and communications system (PACS) used to store patient diagnostic imaging. Cats that received a CT scan during the years 2009–2018 were evaluated for inclusion. Exclusion criteria included maxillofacial trauma, tumors changing osseous architecture of the bones of the skull and patients without permanent dentition. Patient age, sex, weight and breed were recorded for each CT study. Patients were identified as either brachycephalic or mesaticephalic based on the breed recorded in the radiology records.

Transverse slices of the heads were obtained using a helical, 16-slice CT scanner. The settings were 120kVp, 275 mAs and 768 matrix^1^. Images were acquired in 2 mm transverse slices pre and post contrast injection and reconstructed into 2 mm standard and 1 mm bone algorithms. Three-dimensional and multiplanar (transverse, sagittal, and dorsal) reconstructions were created for each study on the same image viewing software^2^ by one of the authors (LD). All reconstructed images came from the pre-contrast 1 mm bone algorithms. A single image of the left and right sagittal view that best demonstrated the distance from the caudal aspect of the infraorbital (IFR) canal to the closest tangential point of the globe of the eye were selected for each cat (LD).

**Figure 1:**
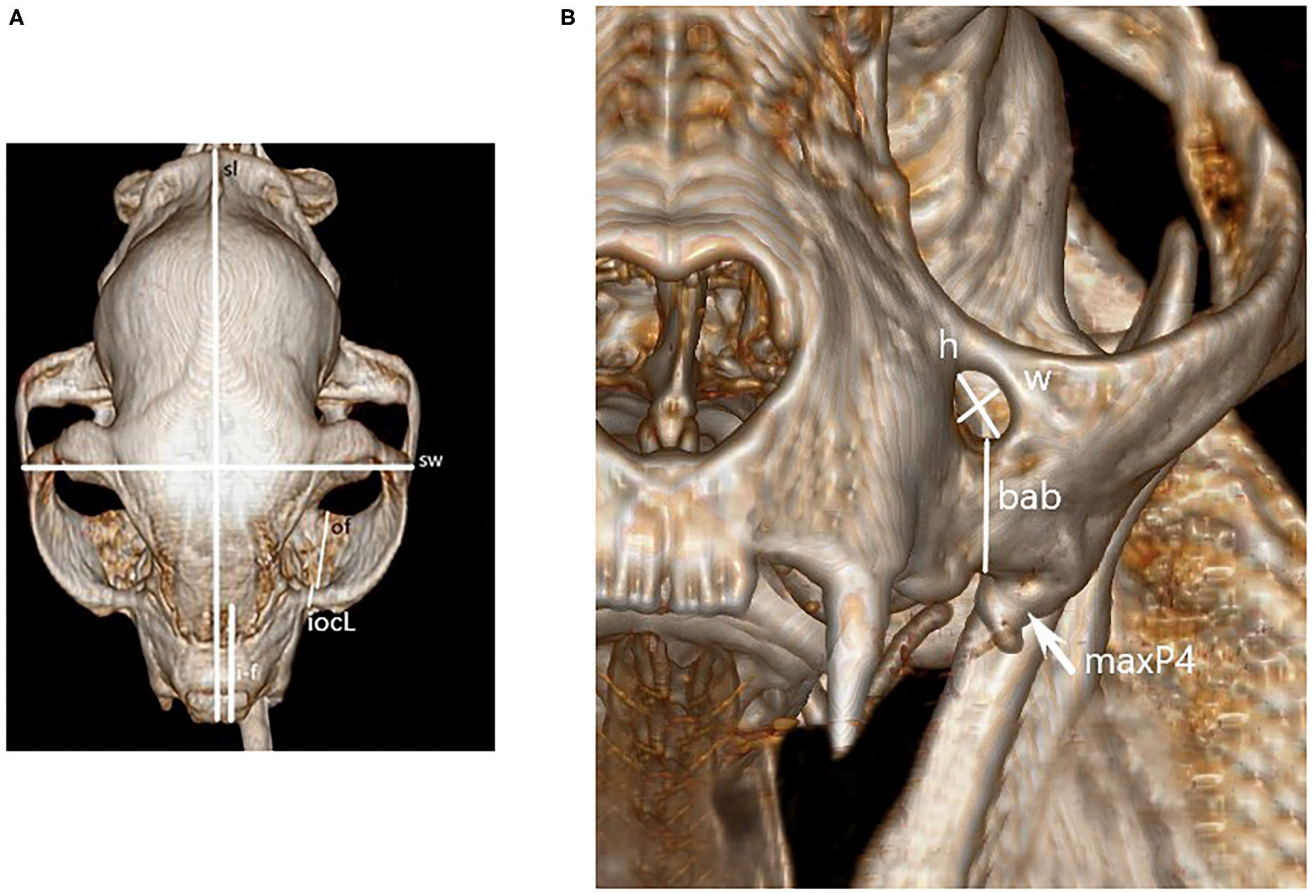
Dorsal **(A)** and mesial **(B)** recon image of a cat skull used for obtaining skull measurements. Figure key: skull length (sl), skull width (sw), distance from rostral incisive bone to rostral IOF (i-f), length of IOC's (iocL), length of the bony orbit floor (of), height (h) and width (w), and buccal alveolar bone at the fourth premolar crown-root junction (bab). The left maxillary 4th premolar is also marked for reference (maxP4).

**Figure 2:**
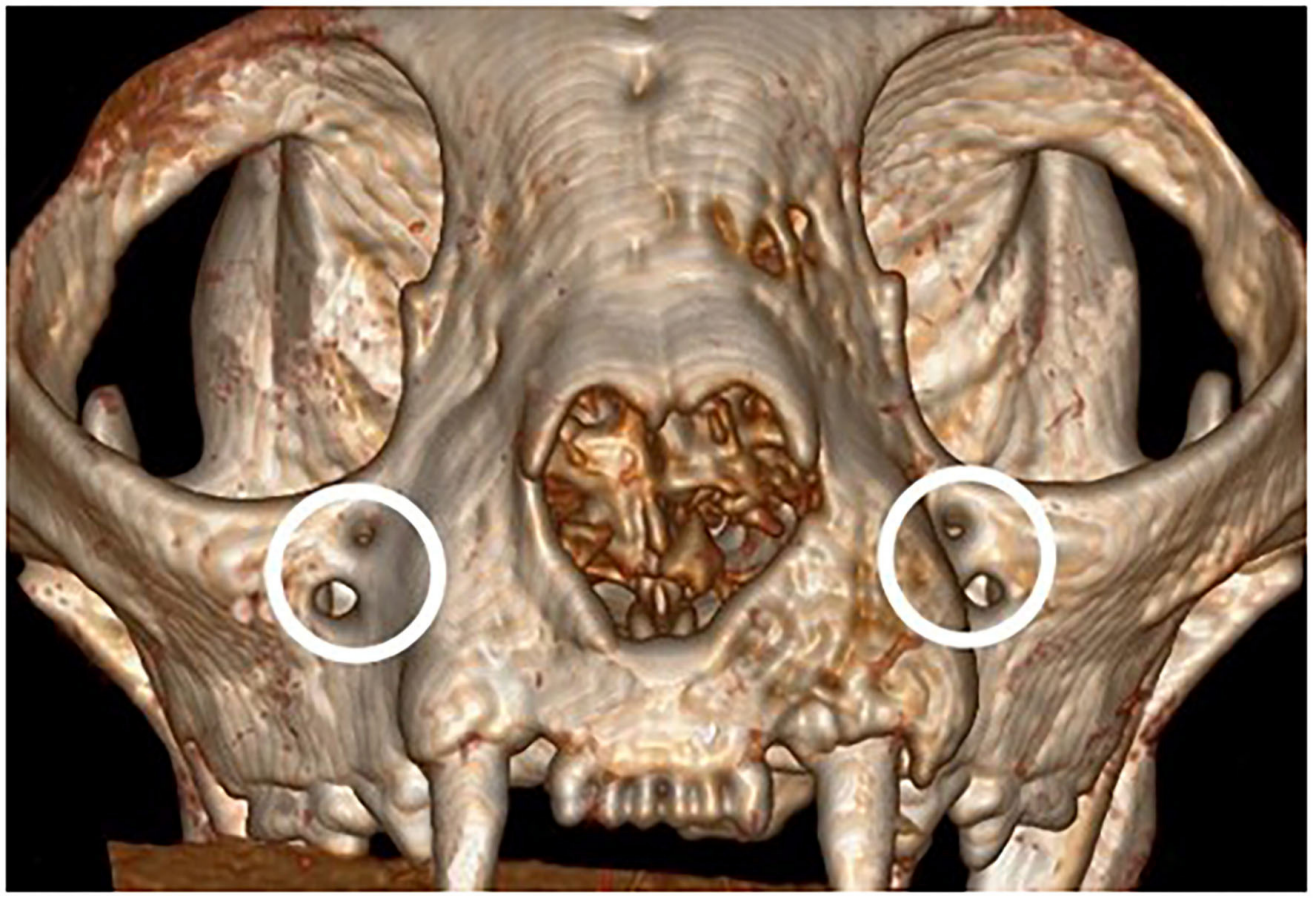
Mesial reconstructed view, displaying redundant IFR foramina.

Once all necessary imaging was compiled, several measurements were acquired. Prior to measurement all images were calibrated from pixels to millimeters, and three independent evaluators (LD, NH, JR) obtained the following measurements utilizing the same software program, computer, and computer screen^3^. Figures 1, 2 identify the location of each measurement. The following single measurements were obtained from the dorsal view of the three-dimensional reconstruction: skull length, skull width, and distance between the rostral incisive bone to the rostral infraorbital foramen (IOF). In addition, the following bilateral measurements were obtained from the same view: infraorbitalcanal (IOC) length and distance from the caudal aspect of the IOC (also known as the maxillary foramen, or “MF”) to the most caudal edge of maxillary bone in the orbit. The following bilateral measurements were obtained from the rostral view of the three-dimensional reconstruction: IOF height and width and the distance between the ventral IOC and the buccal alveolar bone of the fourth premolar tooth at the crown-root junction. The shape of IOF was assigned as round or oval based on the difference in the height and width measurements. If there was a >2 mm difference in height vs. width, it was oval. Using the left and right pre-selected sagittal CT images, the distance between the caudal aspect of MF and the closest tangential point of the globe of the eye was obtained.

Once all observer data were collected, individual reviewer measurements were averaged and analyzed for significance. When measurements differed between observers the statistical test was adjusted to minimize measurer variability. This ensured accurate averages between observers. The data were evaluated for normality. If the data did not meet normality, they was converted into log scale to decrease the scale of the data to try and find normality. This conversion was done prior to building a linear mixed model to compare the parameters between brachycephalic and mesaticephalic cats. The mixed model considered multiple measurements from the same cat evaluated by three different observers. Multiple comparisons were adjusted using a Tukey approach and adjusted means were reported. Age and weight were compared between brachycephalic and mesaticephalic cats using a two-sample Wilcoxon non-parametric test. The association between the two categorical variables were evaluated using a Chi-square or a Fisher's exact test. A *p*-value of 0.05 was used to evaluate statistical significance. The same statistical software was used for all statistical analyses^4^.

## Results

There were no significant differences in reported age, weight and sex of the cats and brachycephalic and mesaticephalic groups. The population of evaluated cats was 13% (6) brachycephalic and 87% (36) mesaticephalic. The mean age of the patients was 15.5 years (std ± 4.52) with a range of 3.5 to 23 years. The mean weight was 4.7 kg (std ± 1.97) with a range of 1.95 to 11.6 kg. There were 18 females (42%) and 28 males (67%). The most common breeds were domestic longhair and shorthair, together making up 33 (79%) patients. Four (67%) of the brachycephalic skulls were found to have redundant foramina, with two smaller foramina instead of a single foramen (Figure 2). All measurements are shown in Tables 1, 2.

**Table 1:** Anatomic skull measurements of the brachycephalic patients.

**Measurement**	**Mean**	**Minimum**	**Maximum**	**Standard**
	**(mm)**	**(mm)**	**(mm)**	**deviation**
Skull length^*^	89.1	71.6	108.8	13.3
Skull width^*^	70.1	65.2	74.5	2.7
L IOC length	4.4	2.9	7.2	1.1
R IOC length	4.4	2.3	7.6	1.1
L IOF height^*^	4.7	3.4	6.4	0.7
R IOF height	4.3	3.2	5.4	0.6
L IOF width	2.7	1.5	4.4	0.7
R IOF width^*^	2.3	1.1	3.7	0.8
Distance from rostral incisive bone to rostral entrance of IOC^*^	18.0	6.5	39.4	9.0
L distance from MF to eyeball^*^	3.1	1	5	1.2
R distance from MF to eyeball^*^	3.3	1.6	5.1	1.0
L buccal alveolar bone at the fourth premolar crown-root junction	5.9	3.9	6.9	1.0
R buccal alveolar bone at the fourth premolar crown-root junction^*^	5.8	4	7.3	1.3
L orbit floor length^*^	11.9	7.8	16.5	2.2
R orbit floor length^*^	12	8.2	16.4	2.2

* *measurements that were significantly different from mesaticephalic skulls have been starred*.

**Table 2:** Anatomic skull measurements of mesaticephalic patients.

**Measurement**	**Mean**	**Minimum**	**Maximum**	**Standard**
	**(mm)**	**(mm)**	**(mm)**	**deviation**
Skull length^*^	99.5	75.8	114.7	7.0
Skull width^*^	67.8	58.1	82	4.6
L IOC length	4.6	2.7	7.8	0.9
R IOC length	4.5	3	6.8	0.9
L IOF height^*^	4.2	1.4	5.9	0.8
R IOF height	4.1	1.6	6.1	0.9
L IOF width	2.9	0.9	6.8	0.8
R IOF width^*^	2.9	1.3	6.3	0.9
Distance from rostral incisive bone to rostral entrance of IOC^*^	23.0	11.5	39.2	5.5
L distance from MF to eyeball^*^	4.0	1.2	6.7	1.0
R distance from MF to eyeball^*^	4.1	1.3	6.4	0.9
L buccal alveolar bone at the fourth premolar crown-root junction^*^	7.1	3.5	10.1	1.2
R buccal alveolar bone at the fourth premolar crown-root junction^*^	7.1	3.7	10.5	1.4
L orbit floor length^*^	13.5	8.9	21.1	2.6
R orbit floor length^*^	13.5	7.8	22.1	2.5

* *measurements that were significantly different from brachycephalic skulls have been starred*.

Brachycephalic cats had a significantly shorter skull length of 89.1 mm (std ± 13.26) compared to mesaticephalic cats 99.5 mm (std ± 6.95) (*p* < 0.001). Brachycephalic cats had a significantly wider skull of 70 mm (std ± 2.7) compared to mesaticephalic cats 67.8 mm (std ± 4.6) (*p* = 0.037). There was a significant difference in the proportion of round and oval foramina shape between brachycephalic and mesaticephalic cats (*p* < 0.001). Among brachycephalic cats, 66% had oval foramina, whereas only 22% of mesaticephalic cats had oval foramina. There was significant difference in the position of the infraorbital foramen between brachycephalic and mesaticephalic cats (*p* < 0.001). The distance from the rostral aspect of the incisive bone to the rostral IFR foramen in brachycephalic cats was 18 mm (std ± 9.0) compared to mesaticephalic cats 23 mm (std ± 5.5). On the sagittal view, in brachycephalic cats, the left and right distances between the maxillary foramen to the ventral eye were 3.1 mm (std ± 1.2) and 3.3 mm (std ± 1), respectively. For mesaticephalic cats, the left and right distances were 4 mm (std ± 1) and 4.1 mm (std ± 0.9), respectively. The distance from the maxillary foramen to the ventral eyeball in brachycephalic cats was significantly shorter compared to mesaticephalic cats' distance (*p* < 0.001). Both left and right sided distances from ventral IFR canal to buccal alveolar bone at the crown-root junction of the fourth premolar were significantly shorter among brachycephalic skulls compared to mesaticephalic skulls (left, *p* < 0.001; right, *p* < 0.001). In brachycephalic cats, these values were 5.9 mm (std ± 1) on the left and 5.8 mm (std ± 1.3) on the right. In mesaticephalic cats, the values were 7.1 mm (std ± 1.2) on the left and 7.1 mm (std ± 1.4) on the right. Across all skull types with edentulous maxillae, there was a significantly shorter distance between the ventral IFR foramen and the buccal alveolar bone of the fourth premolar (*p* < 0.001). There was significant difference between brachycephalic and mesaticephalic cats in their maxillary length on the orbital floor (left, *p* = 0.013; right, *p* = 0.026). The lengths in brachycephalic cats were 11.9 mm (std ± 2.2) on the left and 12 mm (std ± 2) on the right, and mesaticephalic cats measured 13.5 mm (std ± 2.6) on the left and 13.5 (std ± 2.5) on the right.

## Discussion

Our findings demonstrate that there are important variations in the individual anatomic size, shape, and positioning of the IOF and IOC in cats and that there are significant differences between brachycephalic and mesaticephalic feline skulls. In mesaticephalic cats, evaluation of the standard deviation and range demonstrate that even cats of similar size and skull shape can have variations in size and length of the IOF and IOC and the distance of the MF to the ventral globe of the eye. This becomes even more critical in brachycephalic cats as the IOF is smaller, possibly even bifurcated, and the eye is closer to the MF.

Several of the variabilities found between mesaticephalic and brachycephalic cats may directly affect the administration of maxillary nerve regional block via the IOC. The first of these is the presence of redundant foramina in four of the brachycephalic cats examined. It is not known which of the foramen contained the infraorbital nerve, or if the nerve had additional branches that would affect the outcome of the nerve block. Clinicians should be prepared to encounter bifurcated IOF in brachycephalic cats as repeated injection attempts and unsuccessful needle placement could result in significant neurovascular injury and ineffective regional anesthesia.

Although not the focus of the study, the decreasing distance between the alveolar crest to the IOF in cats with edentulous maxillae is an important finding. If a clinician is using the alveolar crest as one of the landmarks for placement of a needle, then knowledge of the decrease in height in edentulous cats is important. In addition, the knowledge of the proximity of the IOF and infraorbital nerve to the alveolar crest in edentulous cats could prevent iatrogenic damage to the neurovascular structures if oral surgery were required in that region. Finally, recognizing that cats with teeth have significantly variable distances from the alveolar crest to the ventral border of the IOF (3.9–7.3 mm in brachycephalic cats and 3.5–10 mm in mesaticephalic cats) is critical when making incisions for mucogingival flaps.

The fact that the length of the IOC did not differ between the mesaticephalic and brachycephalic cats was surprising. While the length of the skulls was significantly different between the skull types, this distance remained similar. The average length of the IOC in mesaticephalic cats was 4.6 mm and in brachycephalic cats 4.4 mm. Because of this length, needle placement deep into the IOC using the infraorbital approach, should be avoided for both brachycephalic and mesaticephalic cats. This is especially critical in brachycephalic cats because the distance between the MF and the ventral aspect of the eyeball is significantly shorter, 3.19 mm compared to 4.05 mm in mesaticephalic cats. Ocular trauma and injection into the eyeball has been reported as complication of the infraorbital nerve block, so knowledge of these anatomical variabilities is critical when utilizing this technique (13–15). Based on these results, it appears that brachycephalic cats may be at a higher risk for ocular injection than mesaticephalic cats when using the infraorbital approach.

One limitation of this study was that due to the retrospective nature of the images, 2 mm slices had been used to obtain the original scans. It would be interesting to repeat the study with either 1 mm slices, or with 0.3 voxels and cone beam computed tomography.

This study demonstrated significant anatomic variability between individual cats, as well as between brachycephalic and mesaticephalic skull types, that had not been previously described in the veterinary literature. Proof of these anatomic differences will direct areas of future study and help determine whether there is a difference in the injectate distribution between brachycephalic and mesaticephalic skull types using the IFR foramen approach to regional anesthesia. In conclusion, there are significant differences between the skull anatomy of both individual cats and mesatacephalic and brachycephalic heads that need to be understood and recognized to increase the safety and efficacy of regional nerve blocks that anesthetize the IFR and maxillary nerves.

## Data Availability Statement

The original contributions presented in the study are included in the article/supplementary material, further inquiries can be directed to the corresponding author/s.

## Ethics Statement

Ethical review and approval was not required for the animal study because this study was completed exclusively on CT images that were stored within the Institution's radiology database, and so an IACUC was not needed. Written informed consent for participation was not obtained from the owners because this study was completed exclusively on CT images that were stored within the Institution's radiology database, so no additional permission was needed.

## Author Contributions

All authors listed have made a substantial, direct and intellectual contribution to the work, and approved it for publication.

## Conflict of Interest

The authors declare that the research was conducted in the absence of any commercial or financial relationships that could be construed as a potential conflict of interest.

## Footnotes

^1^Philips Gemini TF BigBore System, Philips Medical Systems, Cleveland, OH.

^2^Intellispace Radiology v. 4.4551.4, Phillips Healthcare Informatics, Inc, Foster City, CA.

^3^hp EliteDisplay E231, 22.5-inch screen, 1,920 × 1,080 resolution, Palo Alto, CA.

^4^SAS v9.4, SAS Institute Inc., Cary, NC.
